# The Usefulness of Magnetic Resonance Imaging of the Cardiovascular System in the Diagnostic Work-Up of Patients With Turner Syndrome

**DOI:** 10.3389/fendo.2018.00609

**Published:** 2018-10-16

**Authors:** Monika Obara-Moszynska, Justyna Rajewska-Tabor, Szymon Rozmiarek, Katarzyna Karmelita-Katulska, Anna Kociemba, Barbara Rabska-Pietrzak, Magdalena Janus, Andrzej Siniawski, Bartlomiej Mrozinski, Agnieszka Graczyk-Szuster, Marek Niedziela, Malgorzata Pyda

**Affiliations:** ^1^Department of Pediatric Endocrinology and Rheumatology, Poznan University of Medical Sciences, Poznan, Poland; ^2^Cardiac Magnetic Resonance Unit, First Department of Cardiology, Poznan University of Medical Sciences, Poznan, Poland; ^3^Department of Neuroradiology, Poznan University of Medical Sciences, Poznan, Poland; ^4^Department of Pediatric Cardiology, Nephrology and Hypertension, Poznan University of Medical Sciences, Poznan, Poland

**Keywords:** Turner syndrome, girls, MRI, cardiovascular anomalies, magnetic resonance angiography, cardiac magnetic resonance imaging

## Abstract

Cardiovascular defects occur in 50% of patients with Turner syndrome (TS). The aim of the study was to estimate the usefulness of cardiac magnetic resonance imaging (CMR) and magnetic resonance angiography (angio-MR) as diagnostics in children and adolescents with TS. Forty-one females with TS, aged 13.9 ± 2.2 years, were studied. CMR was performed in 39 patients and angio-MR in 36. Echocardiography was performed in all patients. The most frequent anomalies diagnosed on CMR and angio-MR were as follows: elongation of the ascending aorta (AA) and aortic arch, present in 16 patients (45.7%), a bicuspid aortic valve (BAV), present in 16 patients (41.0%), and partial anomalous pulmonary venous return (PAPVR), present in six patients (17.1%). Aortic dilatation (*Z*-score > 2) was mostly seen at the sinotubular junction (STJ) (15 patients; 42.8%), the AA (15 patients; 42.8%), the thoracoabdominal aorta at the level of a diaphragm (15 patients; 42.8%), and the transverse segment (14 patients; 40.0%). An aortic size index (ASI) above 2.0 cm/m^2^ was present in six patients (17.1%) and above 2.5 cm/m^2^ in three patients (8.6%). The left ventricular end-diastolic volume (EDV), end-systolic volume (ESV), and stroke volume (SV) were diminished (*Z*-score < −2) in 10 (25.6%), 9 (23.1%), and 8 patients (20.5%), respectively. A webbed neck was correlated with the presence of vascular anomalies (*p* = 0.006). The age and body mass index (BMI) were correlated with the diameter of the aorta. Patients with BAV had a greater aortic diameter at the ascending aorta (AA) segment (*p* = 0.026) than other patients. ASI was correlated with aortic diameter and descending aortic diameter (AD/DD) ratio (*p* = 0.002; *r* = 0.49). There was a significant correlation between the right ventricular (*p* = 0.002, *r* = 0.46) and aortic diameters at the STJ segment (*p* = 0.0047, *r* = 0.48), as measured by echocardiography and CMR. Magnetic resonance can identify cardiovascular anomalies, dilatation of the aorta, pericardial fluid, and functional impairment of the ventricles not detected by echocardiography. BMI, age, BAV, and elongation of the AA influence aortic dilatation. The ASI and AD/DD ratio are important markers of aortic dilatation. The performed diagnostics did not indicate a negative influence of GH treatment on the cardiovascular system.

## Introduction

Congenital and acquired cardiovascular disease occurs in almost 50% of patients with Turner syndrome (TS) (1) and are the major cause of death in TS, mostly due to dissection of the aorta (2). The incidence of aortic dissection is increased 100 times in TS and is responsible for 2–8% of premature deaths (2–5). Therefore, the evaluation of the cardiovascular system is an important element of the diagnosis in TS. It is essential in terms of the safety aspects of recombinant growth hormone (rGH) treatment and pregnancy planning.

The proven risk factors for aortic dissection are as follows: aortic dilation, bicuspid aortic valve (BAV), aortic coarctation, karyotype 45X, arterial hypertension, and pregnancy (5–9).

Currently, echocardiography is the most popular screening examination for cardiac anomalies in patients with TS. However, it can miss some cardiovascular anomalies (10, 11). Assessment of the aortic arch and descending aorta on echocardiography can be limited by abnormalities of the chest wall and a poor acoustic window (5, 6, 12). Assessment of the aorta is essential due to arteriopathies of aortic arch and descending aorta (7, 13) because ~20% of aortic dissections occur in the descending aorta (5, 6). The aortic diameter is a risk factor for aortic dissection and can be monitored by cardiac magnetic resonance imaging (CMR).

Cardiovascular magnetic resonance imaging allows a noninvasive assessment of whole aorta without ionizing radiation, enabling recognition of clinically, and sonographically silent anomalies (7, 11). Magnetic resonance, in particular, offers information that is difficult to obtain from other imaging modalities such as complex congenital cardiovascular anomalies and quantitate aspects of regional ventricular function. The guidelines for the care of girls and women with TS recommend that CMR should be used as a screening tool in all children with TS at an age when it can be performed without sedation, even if echocardiography did not reveal any abnormalities (14, 15).

The aortic diameter is determined by age and body size (16), so aortic dimensions must be adjusted for body surface area (BSA). The aortic size index (ASI) calculated as the ratio between the ascending aortic diameter and the BSA is currently commonly used in clinical practice (2, 17). TS patients with an ASI >2 cm/m^2^ are at high risk for aortic dissection, and those with an ASI >2.5 cm/m^2^ are at a very high risk (2, 17).

There is no consensus on whether dilatation of the aorta may occur during early childhood and which CMR parameters can predict dissection of aorta. There is still discussion about the role of echocardiography in the CMR era for the diagnosis of cardiovascular anomalies in TS.

**The aim** of the present study is to evaluate the usefulness of CMR and 3D dynamic magnetic resonance angiography (angio-MR) in the diagnosis of anomalies of the aorta and other vessels and to establish risk factors for aortic dilatation in TS patients. The other aims are to compare the usefulness of CMR and echocardiography in TS and to estimate the risk factors of aortic dissection, the correlations of aortic diameter with several clinical factors (age, BMI, karyotype) and CMR parameters.

## Patients and methods

Forty-one patients with recognized TS, aged 13.9 ± 2.2 years, were studied. The exclusion criteria were as follows: lack of informed consent, contraindications for magnetic resonance studies, or a lack of cooperation during the CMR study. An ethical review process was not required for this study because it utilizes the standard diagnostic tests for TS. Before CMR and angio-MR, each patient signed the informed consent. Each patient had echocardiography performed before CMR and angio-MR performed ~6 months after CMR. CMR was performed in 39 patients (95.1%), and angio-MR was performed in 36 (87.8%) patients. One patient did not have angio-MR with contrast, and aortic measurements were not possible. In 34 (82.9%) TS patients, both CMR and angio-MR were performed.

Height and BMI centiles were calculated with the OLAF calculator using normal Polish ranges (18). The height standard deviation score (HtSDS) was calculated using the same ranges. Overweight and obesity were diagnosed according to the International Obesity Task Force Criteria (19). Arterial hypertension was diagnosed when systolic and diastolic blood pressure exceeded or were equal to the 95th centile for age and/or height for the Polish population (18).

Karyotype was established due to conventional cytogenetics analysis by peripheral lymphocytes (20–100 metaphase plates) evaluation.

Twenty patients (48.8%) had a webbed neck, which is defined as redundant cervical skin folds arcing out from mastoid at the level of ear lobe to the acromion. Hypothyroidism was diagnosed in 20 patients (48.8%) and treated with L-thyroxine. In 18 patients, autoimmunologic inflammation of the thyroid gland was detected. Growth hormone therapy was induced in 36 patients (87.8%). The average dose was 0.025–0.055 mg per kg body weight per day. Estrogen replacement therapy was administered to 25 patients (61.0%). Clinical data of the studied population are presented in Table 1.

**Table 1:** Clinical characteristics of the study group.

	***n* = 41**
Age at CMR (years)	13.9 ± 2.2
Age at angio-MR (years)	14.6 ± 2.2
Height SDS	−2.16 ± 1.1
BMI (centile)	69.7 ± 25.2
Previous cardiac surgery (*n* %)	6, 14.6%
Karyotype 45,X (*n* %)	18, 44%
Webbed neck (*n* %)	20, 48.8%
Arterial hypertension (*n* %)	3, 7.32%
Hypothyroidism (*n* %)	20, 48.8%
Growth hormone therapy (*n* %)	36, 87.8%
Estrogen replacement therapy (*n* %)	25, 61%

Transthoracic 2D and Doppler echocardiography were performed using a Vivid E9 (GE, Little Chalfont). The standard views and measurements were obtained according to the ESC guidelines. Aortic diameters were measured using an edge-to-edge technique at the aortic ring, sinotubular junction (STJ) and sinus of Valsalva.

CMR and 3D dynamic MR angiography were performed with a 1.5-T scanner (Siemens, Avanto) with the use of a matrix coil for body and cardiac applications combined with a spinal coil. All sequences were performed with ECG triggering during breath-hold. Cardiac MRI included the following sequences: anatomical imaging, ventricular volume and functional assessment and phase-contrast flow quantification. Anatomical imaging was obtained with an echo-planar fast-spin echo sequence (HASTE—Half-Fourier Acquisition Single-shot Turbo spin Echo) in three orthogonal planes (axial, coronal, and transverse). The imaging parameters were as follows: TR/TE 2 R-R intervals/27 ms, field of view 380 × 260 mm, slice thickness 8 mm, gap 2 mm, and matrix size 104 × 256. Cine MRI was performed with a steady-state free precession (SSFP) sequence in two-, three-, and four-chamber views. A short-axis stack was obtained from the cardiac base to apex. The typical parameters were TR/TE 55.88/1.07 ms, field of view 380 × 310 mm, slice thickness, 8 mm, gap 2 mm, matrix size 109 × 192, and in-plane resolution 2.8 × 2.0 mm. The volumetric method was used to evaluate the left ventricular end-diastolic volume (EDV), end-systolic volume (ESV), stroke volume (SV), ejection fraction (EF), and mass. The diameters of the right ventricle and the left ventricle (LV) in diastole, the diameter of the left atrium in systole, and the thickness of the interventricular septum and inferior wall were also measured.

Flow imaging was performed with a free breathing ECG-gated flow-sensitive sequence. A through-plane phase-contrast gradient-echo sequence was performed at the level of the AA above the aortic and pulmonary valves, and the imaging parameters were as follows: velocity encoding 150 m/s for the aorta and 120 m/s for the pulmonary artery, TR/TE 29.90/2.18 ms, field of view 380 × 285 mm, slice thickness 5 mm, gap 1 mm, matrix size 192 × 256, and in-plane resolution 1.5 × 1.5 mm. Phase-contrast flow quantification was used to assess the ratio of pulmonary flow (Qp) to systemic flow (Qs) ratio and aortic and pulmonary regurgitation.

**Figure 1:**
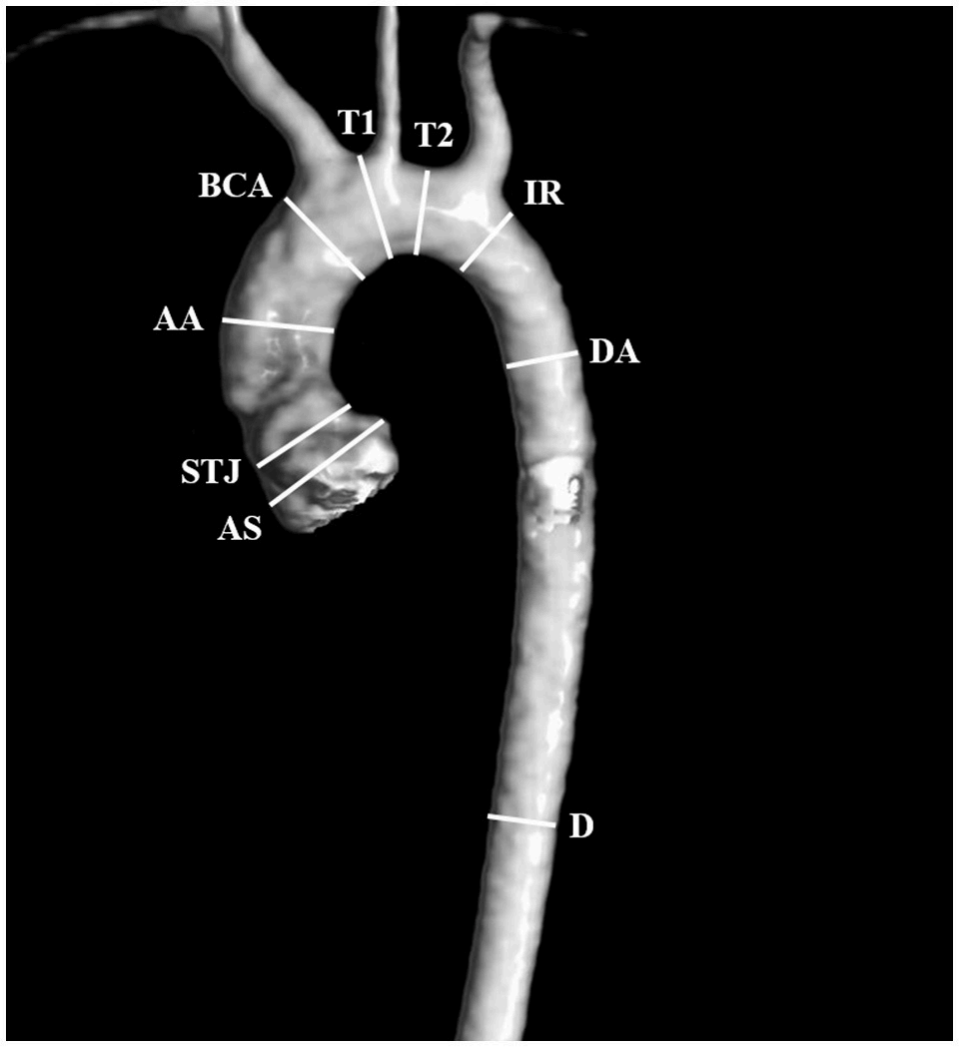
Locations of aortic diameter measurements: aortic sinus (AS), sinotubular junction (STJ), ascending aorta (AA), at the origin of the brachiocephalic artery (BCA), first transverse segment (T1), second transverse segment (T2), isthmic region (IR), descending aorta (DA), and the thoracoabdominal aorta at the level of the diaphragm (D).

**Figure 2:**
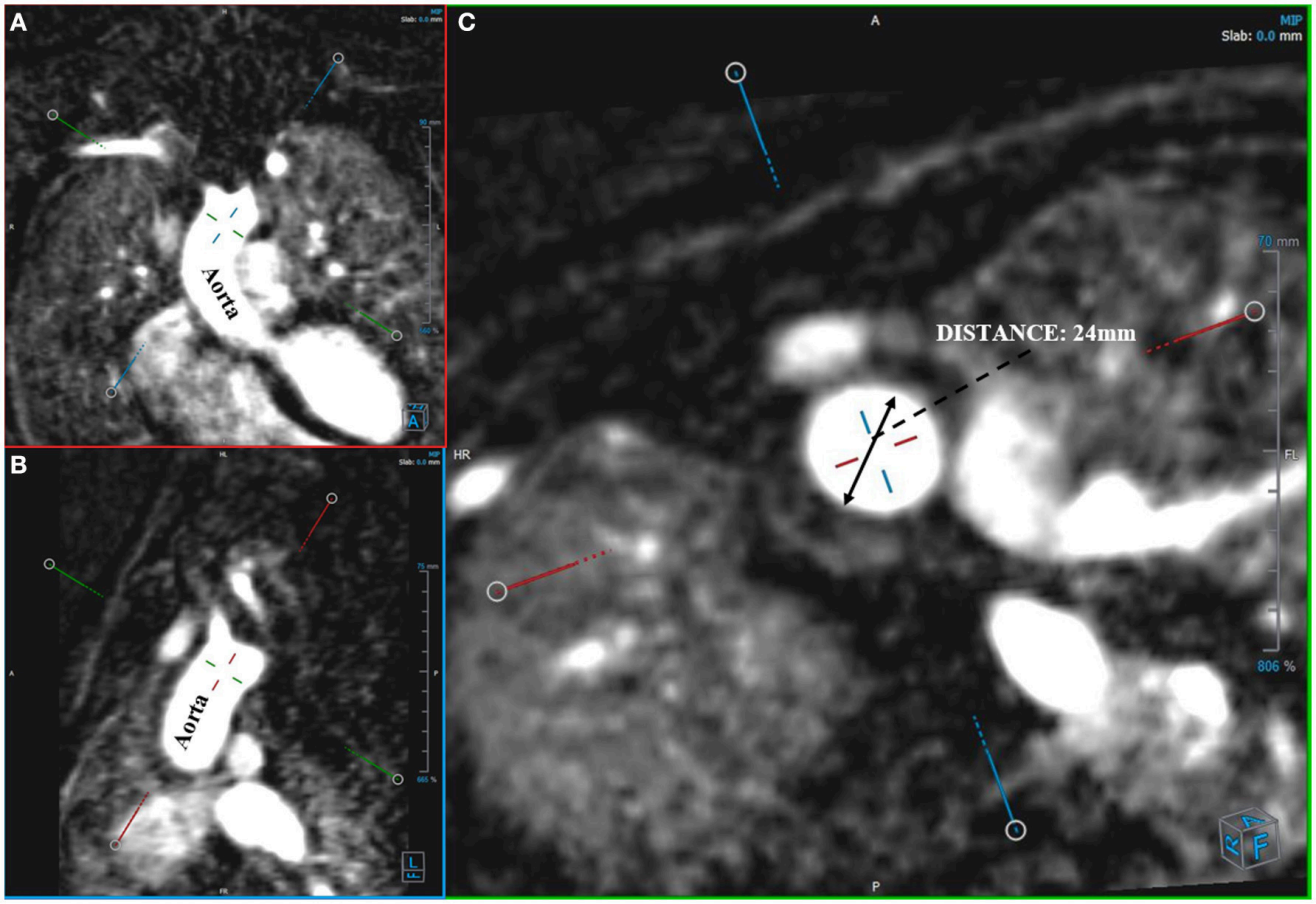
Measurement of aortic diameter. Angio-MR **(A,B)**, -perpendicular planes **(C)**, -diameter of the ascending aorta in a transverse plane.

**Figure 3:**
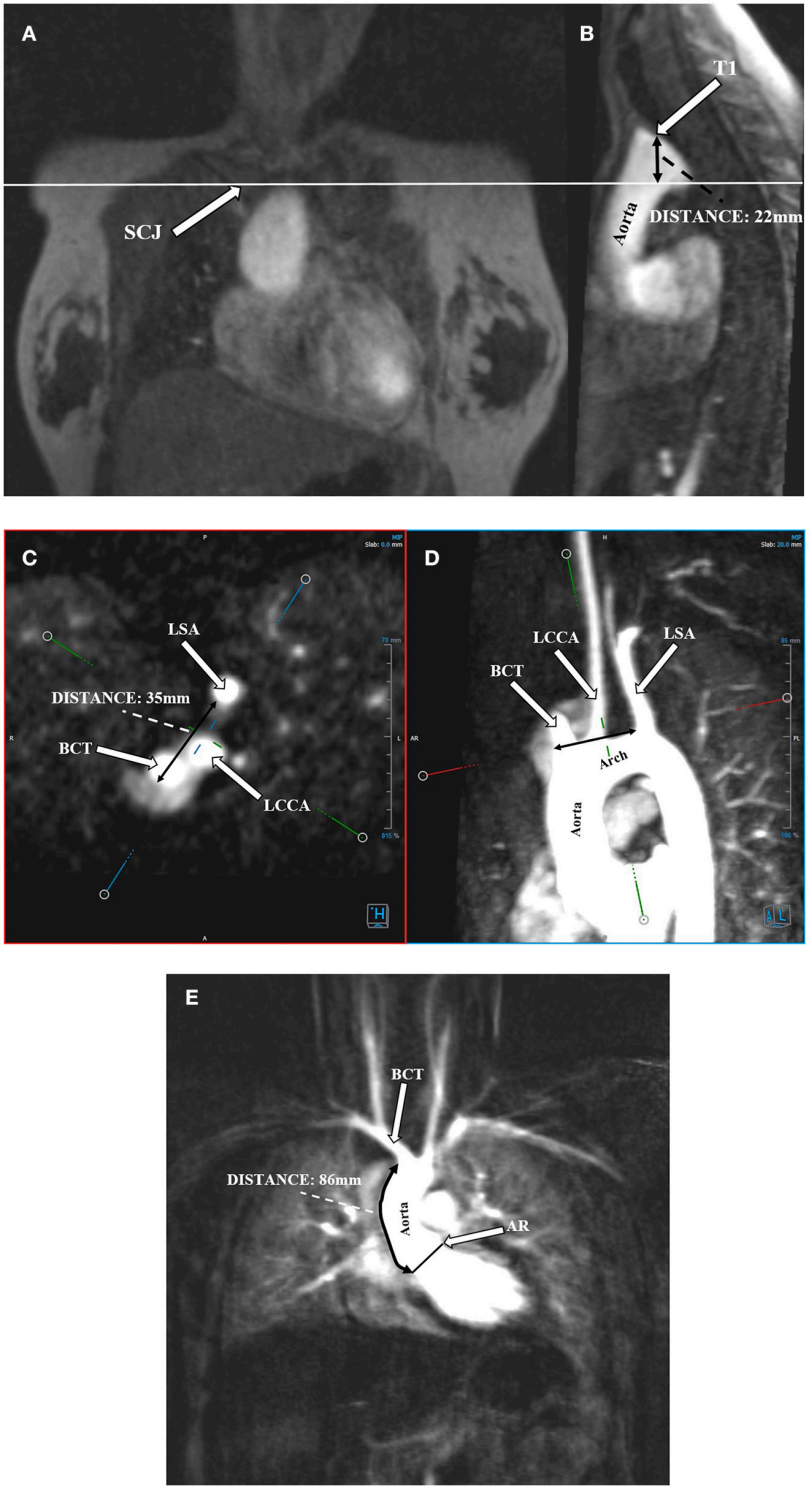
Protrusion of the aortic arch above the sternoclavicular joint. Distance between the highest point of the first transverse segment (T1) and the sternoclavicular joint (SCJ). **(A)** Angio-MR, coronal plane. SCJ (arrow); the perpendicular line shows the level of the SCJ. **(B)** Angio-MR, sagittal plane, showing the distance between the highest point of T1 and SCJ. Measurement the length of the aortic arch between the brachiocephalic trunk (BCT) and the left subclavian artery (LSA). **(C)** Measurements of the BCT, LCCA (left common carotid artery), and LSA were made on Angio-MR in a transverse plane. **(D)** Angio-MR of the aorta showing the spatial orientation of the arch and its branches (BCT, LCCA, LSA) and the measurement locations along the length of the aortic arch (black arrow). **(E)** Elongation of the ascending aorta. Distance between the aortic ring (AR) and the brachiocephalic trunk (BCT). Angio-MR, coronal plane.

Angio-MR was performed with the use of the dynamic *Time-resolved Angiography With Interleaved Stochastic Trajectories* (TWIST) after the administration of a contrast agent (0.1 mmol/kg) followed immediately by a 20 ml saline flush. The temporal resolution varied between 3 and 5 s, with an overall sequence time of ~100 s. The time of contrast injection was calculated following the administration of 1 ml of contrast bolus. The typical sequence parameters were: TR/TE 2.3/0.87 ms, field of view, 500 × 310 mm, slice thickness 1.5 mm, gap 0 mm, matrix size 384 × 224, and in-plane resolution 1.40 × 1.30 mm. The TWIST sequence was used for the evaluation of vascular anomalies and the following measurements. The aortic diameter was measured at nine levels including the aortic sinus (AS), STJ, AA, at the origin of the brachiocephalic artery (BCA), first transverse segment (T1), second transverse segment (T2), isthmic region (IR), descending aorta (DA), and thoracoabdominal aorta at the level of diaphragm (D) (Figures 1, 2A–C). Distances between the first transverse segment (T1) and the sternoclavicular joint, the length of the aortic arch between the BCA and the left subclavian artery, and elongation of the AA measured between the aortic ring and the BCA were also estimated (Figures 3A–E). All measurements were obtained with dedicated the software Medis Suite MR 3.0.

Measurements of aortic diameters and ventricular volumes were standardized by body surface area. The results for the aorta were compared to ranges developed by Kaiser et al. (20). A standardized Z-score for aortic diameter at each segment was calculated with an electronic calculator developed by Kaiser et al. (20), in which the diameter of each segment of the aorta and BSA were used. Ventricular volumes and myocardial mass were compared to ranges estimated by Buechel et al. (21). The ASI, which is defined as the ratio between ascending aortic diameter and the BSA, was calculated. The ratio between the ascending aortic diameter and the descending aortic diameter (AD/DD ratio) was also evaluated.

The calculations were performed using the Statistica 12 program from StatSoft and StaXact from Cytel. An α = 0.05 was assumed as the significance level. The results were considered statistically significant when *p* < α. Continuous variables are shown as the mean ± standard deviation, the minimum and maximum values and the median. Numbers and percentages are given for categorical variables. The normality of the distribution of variables was evaluated using the Shapiro-Wilk test. To compare the variables, Student's *t*-test for unrelated samples was used, if the distribution of the variable was consistent with the normal distribution and the variances were equal; the Mann-Whitney test was used when the variable were not normally distributed. To investigate the relationship between continuous variables, in cases in which both of variables were normally distribution, the Pearson *r* correlation coefficient was calculated, while the Spearman *r* rank correlation coefficient was calculated when the variables were not normally distributed. To test the relationship between categorical variables, the chi-square test, Fisher's exact test or the Fisher-Freeman-Halt test were used. In the cases of dependency, the odds ratio was calculated along with 95% confidence intervals. To estimate whether there was a statistically significant difference between the measurements obtained using two techniques, Student's *t*-test for related samples or the Wilcoxon test were used.

## Results

In 18/41 (44.0%) patients the 45,X karyotype was detected, and the rest had different mosaic karyotypes. Thirteen patients (13/41; 31.7%) were overweight, and 1 patient (1/41; 2.4%) was obese. Six patients underwent cardiac surgery; five of these patients (5/41; 12.2%) had coarctation of the aorta, and one (1/41; 2.4%) had a patent ductus arteriosus (PDA). Arterial hypertension was diagnosed in three patients (3/41; 7.3%). Details of the clinical characteristics of the patients and results of ECHO, CMR, and angio-MR are presented in Table S1.

**Figure 4:**
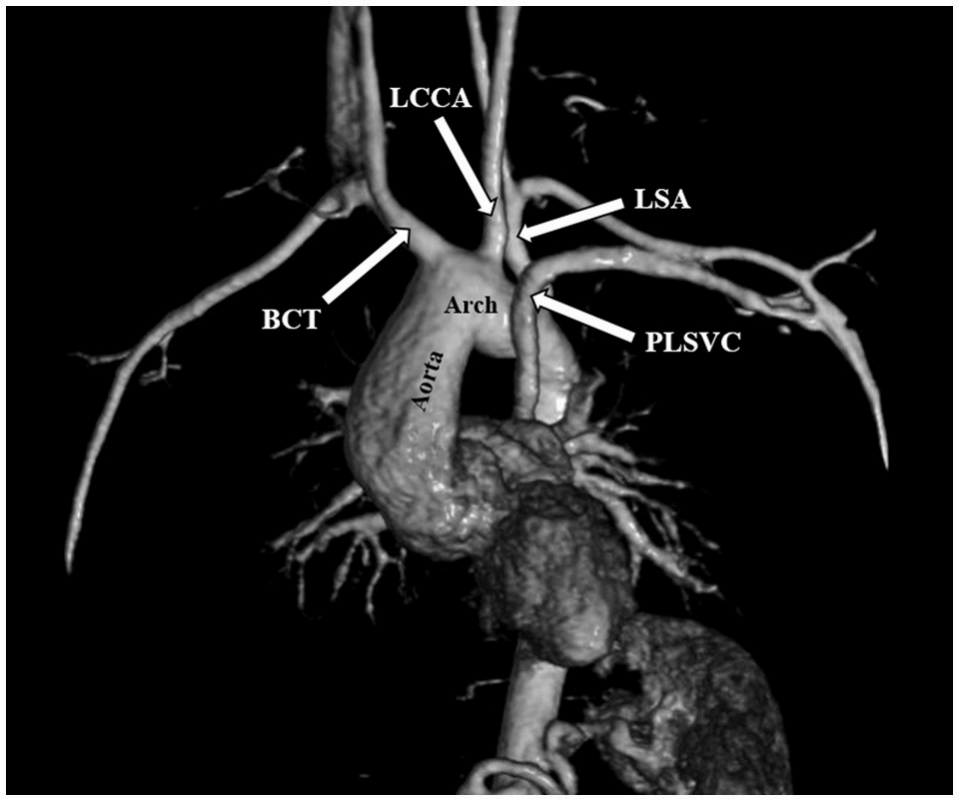
Persistent left superior vena cava (PLSVC). 3D MRA. BCT, brachiocephalic trunk; LCCA, left common carotid artery; LSA, left subclavian artery.

**Figure 5:**
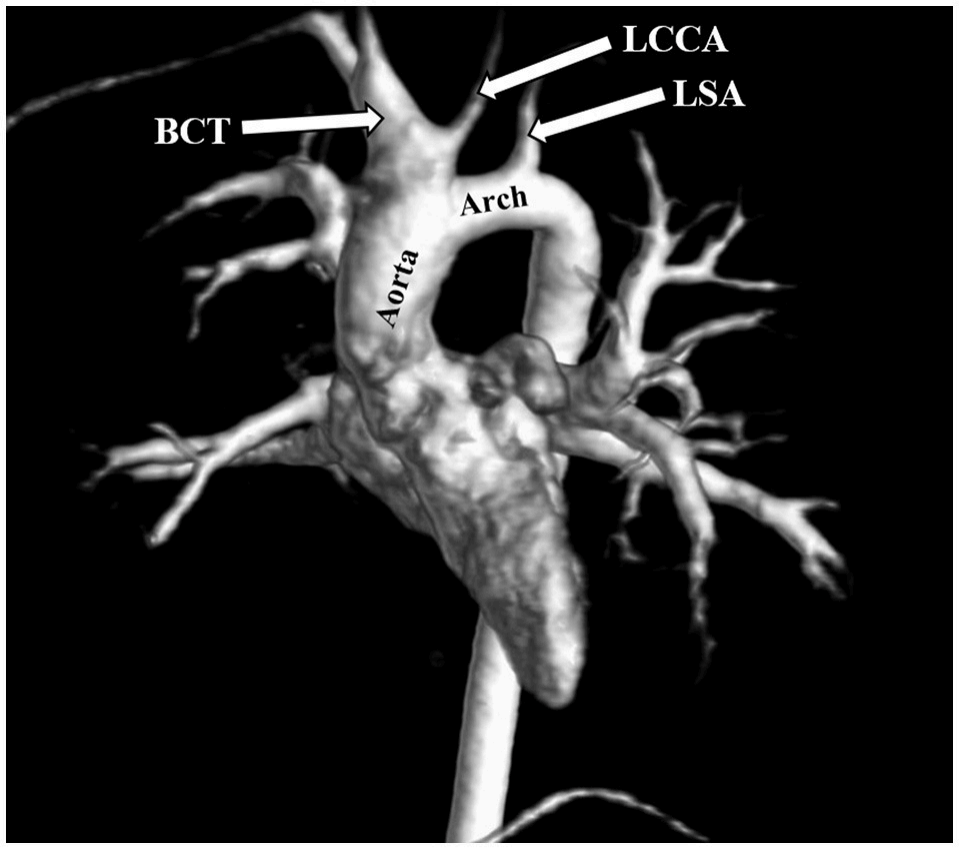
Bovine arch. Left common carotid artery (LCCA) arising from the brachiocephalic trunk (BCT). 3D MRA. LSA, left subclavian artery.

The most frequent anomalies diagnosed on CMR and angio-MR were as follows: elongation of the AA and aortic arch in 16/35 (45.7%) patients, BAV in 16/39 patients (41.0%), partial anomalous pulmonary venous return (PAPVR) in 6/35 patients (17.1%), persistent left superior vena cava (PLSVC) in 4/35 patients (11.4%; Figure 4) and bovine arch in 3/35 patients (8.6%; Figure 5; Table 2). Most of these congenital vascular anomalies, except BAV, were missed by echocardiography. Only 3/39 BAV cases (7.7%) were underdiagnosed by echocardiography. In one patient (1/39; 2.6%), BAV was diagnosed on echocardiography but was not confirmed on CMR. In 7/39 patients (17.9%) pericardial fluid seen on CMR was not detected by echocardiography.

**Table 2:** Cardiovascular anomalies in patients with Turner syndrome on angio-MR vs. ECHO.

**Anomaly**	**Angio-MR *n*, %**	**ECHO *n*, %**
Elongation of the ascending aorta and aortic arch	16 (45.7%)	Not detected
Bicuspid aortic valve (BAV)	16 (41.0%)	14 (36.1%)
Partial anomalous pulmonary venous return (PAPVR)	6 (17.1%)	Not detected
Persistent left superior vena cava (PLSVC)	4 (11.4%)	Not detected
Common origin of the left common carotid artery (LCCA) and brachiocephalic trunk (BCT) - bovine arch	3 (8.6%)	Not detected
Aberrant right subclavian artery	2 (5.7%)	Not detected
Right sided aortic arch	1 (2.8%)	Not detected
Anomalous left vertebral artery origin	1 (2.8%)	Not detected
Atrial septal defect (ASD)	1 (2.6%)	Not detected

The mean aortic diameters and number of patients with enlarged aortic segments (*Z*-score >2) are presented in Table 3. Aortic dilatation was most frequently seen at the STJ (15/35 patients, 42.9%), AA (15/35 patients, 42.9%), thoracoabdominal aorta at the level of the diaphragm (15/35 patients, 42.9%), and in the first transverse segment (14/35 patients, 40.0%). The mean ratio between the ascending aortic diameter and the descending aortic diameter (AD/DD ratio) was 1.55 ± 0.21 (range 1.09–2.00). Fifteen patients (15/35; 42.9%) had an AD/DD ratio > 1.5. ASI > 2 cm/m^2^ was present in six patients (6/35; 17.1%), and an ASI > 2.5 cm/m^2^ was present in three patients (3/35; 8.6%; with a maximum of 2.79 cm/m^2^).

**Table 3:** Aortic diameters and the number of patients with dilated aortic segment.

**Segment of measurement**	**Diameter (mean, range) (mm)**	**Aortic diameter index (mean) (mm/m^2^)**	**Number of patients with *Z*-score > 2 (*n*,%)**
Aortic sinus (AS)	27.2 (19.0–44.0)	20.0	12 (34.3)
Sinotubular junction (STJ)	23.5 (15.0–37.0)	17.37	15 (42.9)
Ascending aorta (AA)	24.3 (17–41.0)	17.98	15 (42.9)
Brachiocephalic artery (BCA)	22.3 (16.0–34.0)	16.42	8 (22.9)
First transverse segment (T1)	21.0 (14.0–35.0)	15.40	14 (40.0)
Second transverse segment (T2)	18.8 (13.0–31.0)	13.81	11 (31.0)
Isthmic region (IR)	17.4 (12.0–22.0)	12.81	9 (25.7)
Descending aorta (DA)	17.5 (13.0–29.0)	12.95	7 (20.0)
Thoracoabdominal aorta at the level of the diaphragm (DD)	15.7 (12.0–23.0)	11.63	15 (42.9)

The mean EDV of the left ventricle was 94.29 ml, and the mean EDV index was 68.94 ml/m^2^. Ten patients (10/39; 25.6%) had an EDV *Z*-score < −2, and two patients (2/39; 5.1%) had an EDV *Z*-score > 2. The mean ESV of the left ventricle was 37.13 ml, and the mean ESV index was 27.14 ml/m^2^. Nine patients (9/39; 23.1%) had an ESV *Z*-score < −2, and two patients (2/39; 5.1%) had an ESV *Z*-score > 2. The mean SV was 57.15 ml, and the mean SV index was 41.8 ml/m^2^. Eight patients (8/39; 20.5%) had an SV *Z*-score < −2.

Karyotype 45,X was not associated with an increased prevalence of cardiovascular abnormalities. There was no significant difference in the aortic diameters or *Z*-scores of aortic diameters between patients with or without karyotype 45,X. However, patients with karyotype 45,X more often had an aortic diameter at the T1 segment with a *Z*-score > 2 compared with patients without karyotype 45,X (*p* = 0.023).

The prevalence of cardiovascular anomalies was higher in patients with a webbed neck, especially BAV (*p* = 0.04), PAPVR (*p* = 0.008), and PLSVC (*p* = 0.047). Age was correlated with the diameter of the aorta at the following segments: BCA (*r* = 0.43, *p* = 0.009), T1 (*r* = 0.57, *p* < 0.001), T2 (*r* = 0.61, *p* < 0.001), IR (*r* = 0.69, *p* < 0.001), and DA (*r* = 0.35, *p* = 0.039). BMI was correlated with the diameter of the aorta at BCA (*r* = 0.39, *p* = 0.018), T2 (*r* = 0.36, *p* = 0.032), and DD (*r* = 0.36, *p* = 0.03).

**Figure 6:**
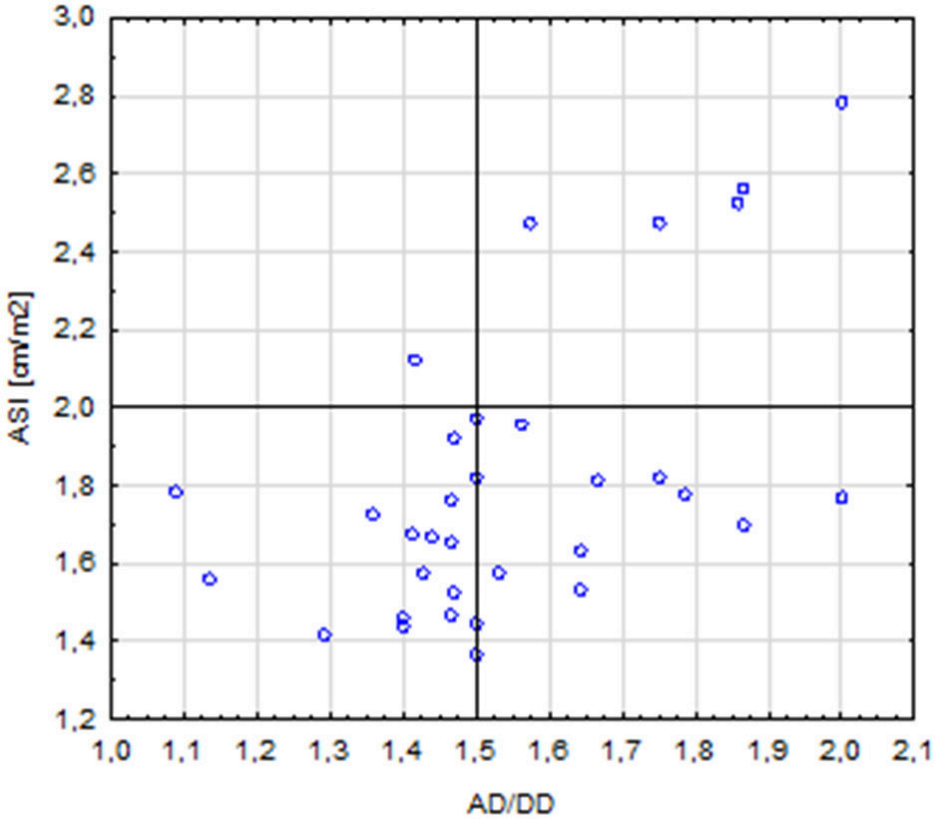
Correlation between the ASI (aortic size index) >2 and AD/DD (aortic ascending/descending diameter) >1.5 (*p* = 0.015).

The length of the AA was correlated with the diameter of the aorta at the following segments: AS (*r* = 0.64, *p* < 0.001), STJ (*r* = 0.51, *p* = 0.001), AA (*r* = 0.44, *p* = 0.008), T1 (*r* = 0.41, *p* = 0.014); it was also correlated with the length of the aortic arch (*r* = 0.59, *p* < 0.001). A total of five patients with corrected coarctation of the aorta had a statistically greater aortic diameter at the STJ (*p* = 0.01) and smaller diameter at the IR (*p* = 0.006). Patients with BAV had significantly bigger aortic diameter at AA (*p* = 0.026). We also found a correlation between the ASI and AD/DD ratio (*r* = 0.49, *p* = 0.002). Patients with an ASI >2 cm/m^2^ more often had an AD/DD ratio >1.5 compared with those with an ASI < 2 cm/m^2^ (*p* = 0.015) (Figure 6). Patients with BCA and a DD *Z*-score >2 had a significantly longer aortic arch than that of other patients (*p* = 0.023 and *p* = 0.047, respectively).

There was agreement of most of the measurements made on echocardiography and CMR, especially the right ventricular diameter (*r* = 0.46, *p* = 0.002) and the aortic diameter at the STJ segment (*r* = 0.48, *p* = 0.005).

## Discussion

The relatively young age of the studied group is exceptional. Most of the recently published studies have evaluated young adults with TS (7, 12, 22, 23).

We report a high prevalence of vascular anomalies diagnosed on angio-MR and CMR. The pathophysiology of cardiovascular abnormalities in TS is still under debate. There is a hypothesis that jugular lymphatic sac obstruction in 45,X fetuses can lead to distended thoracic ducts, compression of the AA, and reduced intracardiac blood flow (24). Reduced blood flow leads to impaired development of aortic valve and arch. Thus, patients with webbed necks are suspected to have cardiovascular defects. The X-chromosome genes that are responsible for congenital heart defects in TS have not been identified yet. Prakash et al. analyzed 454 TS subjects and found that left-sided congenital heart lesions were associated with a reduced dosage of Xp genes and increased dosage of Xq genes (25). They also showed that genome-wide copy number variation is increased in TS, and they identified a common copy number variant (CNV) in chromosome 12p13.31 that is associated with left-sided congenital heart lesions. The CNV contained three protein-coding genes (*SLC2A3, SLC2A14*, and *NANOGP1*) and was previously implicated in congenital heart defects in 22q11 deletion syndrome. Additionally, they identified a subset of rare and recurrent CNVs that are also enriched in asymptomatic BAV cases. There is also a hypothesis that haploinsufficiency of the *FOXC2* gene, which codes a forkhead winged-helix transcription factor, is responsible for cardiovascular defects (2). Several syndromes including congenital heart defects are attributed to X-linked genes. Mutations of the following genes can lead to cardiovascular disease: *Filamin A, EMD* (emerin), *LAMP2* (lysosomal-associated membrane protein 2), *DMD* (dystrophin), *TAZ* (tafazzin), and *VEGF-D* (vascular endothelial growth factor D) (26). Although TS patients have no signs of connective tissue defects, there is a theory that arterial TGF-beta signaling in TS can be disrupted and lead to aneurysm formation and a risk of vascular dissection (27).

In the studied group, karyotype 45,X was not associated with an increased prevalence of cardiovascular abnormalities, and there was no significant difference in the aortic diameters between patients with or without karyotype 45,X. Patients with karyotype 45,X had greater aortic diameter at the T1 segment than patients without karyotype 45,X. It is well known that patients with TS and pure karyotype 45,X are more affected by developmental anomalies including fetal lymphedema, BAV, and aortic coarctation (2). The relationship between karyotype 45,X, BAV, and aortic dilatation were confirmed in a French cohort study (28). However, Cleeman et al. did not find a direct association between karyotype 45,X and the diameter of the aorta (29). The potential reason for cardiovascular anomalies in karyotype 45,X patients is haploinsufficiency for Xp genes (30). It seems that the correlation between cardiac and aortic anomalies and karyotype should be more profound in patients lacking one Xp arm and not those with karyotype 45,X.

A webbed neck, which is a sign of fetal lymphedema, is strongly associated with cardiovascular defects (1, 31) and it is also confirmed by our study. The age of TS patients is also positively correlated with the diameter of the aorta as documented by others (22, 29, 32). Dissection and rupture of the aorta were not seen in our population due to their young age, while in the Danish and Swedish studies, the median age of onset of aortic dissection or rupture in TS patients was found to be 35 years (5). Our study also confirmed that BMI is associated with the dilatation of the aorta. Obesity is a known risk factor for increased aorta diameter, which is associated with arterial stiffness and a greater carotid intima-media thickness (33). Since patients and women with TS have an increased risk of metabolic disturbances and overweight, they should be carefully monitored.

In our study, the frequencies of cardiovascular anomalies such as aortic coarctation (12.2%), elongation of the AA (45.7%), PAPVR (17.1%), PLSVC (11.4%), bovine arch (8.6%), aberrant right subclavian artery (5.7%), and anomalous left vertebral artery origin (2.8%) were similar to those reported in other studies (1, 23). The prevalence of BAV in our group (41%) was relatively higher than that in previous published studies (25–30%) (7, 10, 13). Only Kim et al. (23) reported a similar BAV prevalence (39%). We observed a high percentage (42.9%) of patients with an increased aortic diameter. Aortic dilatation is a known serious risk factor for aortic dissection (8); thus, the diagnosis of aortic dilatation is essential in clinical practice. Aortic dilatation has been reported in 32–42% of patients and women with TS (8, 11, 34, 35). Kim et al. reported aortic dilation at the AS segment in 30% of patients and at the STJ in 26% (23). However, in the Danish study, the diameters of aorta in TS patients were significantly smaller at the aortic arch and descending aorta than those of control subjects (29). The standardization of aortic diameter measurements in TS is crucial because TS patients are usually shorter than the healthy population. Therefore, in our study, the diameters and ventricular volumes were indexed with BSA. Approximately 17.1% of our patients had an ASI >2 cm/m^2^, which is also assumed to be a high-risk factor for aortic dissection (2, 17). Three patients had an ASI >2.5 cm/m^2^, which is an extremely high-risk factor and prompts the need for surgical intervention (8, 26). If the descending aortic diameter is within the normal range, another ratio can be used to estimate the AA dilatation: an AD/DD ratio >1.5 (11). Some studies showed that the ASI is a more reliable parameter in TS than the AD/DD ratio (8). In a study by Matura et al. 33% of women with an ASI >2.5 cm/m^2^ experienced aortic dissection within 3 years, and 3% of those with an AD/DD ratio >1.5 had aortic dissection (8). In our study, the presence of BAV was also associated with a greater diameter of the AA. Isolated BAV is usually associated with larger proximal aortic diameters, apart from normal valve function (36). Identifying BAV in asymptomatic individuals is also important because they are at increased risk for valvular dysfunction, infective endocarditis, and aortic aneurysm (10). In our study, BAV was diagnosed on CMR in three patients (7.7%) but was missed on echocardiography.

The association between elongation of the aorta and aortic dissection is still debated. In our study, elongation of the AA was associated with a greater aorta diameter. Thus, aortic elongation may be a contributing factor for aortic dilatation. A recent study from Germany showed that aortic elongation may play a role in the pathogenesis of aortic dissection (37).

Left ventricular volumes (EDV, ESV, and SV) as evaluated by CMR were decreased in 20–25% of our population. Considering that 87.8% of the studied patients were treated with rGH, the safety of which might be questioned. Neither myocardial hypertrophy nor ventricular dysfunction was found in our population, similar to the findings of previous studies on the safety of rGH treatment (38–40). The results concerning left ventricle volumes are similar to those found by Van den Berg et al. (38) but contrast those of other papers that report comparable LV volumes between healthy controls and TS patients (39, 40). The decreased left ventricle volumes may be explained by the different effects on the growth of various organs as shown in the experimental models of GH and IGF-I deficiency (41, 42). However, a recent echocardiographic study in TS showed no evidence for disproportionate cardiac growth between patients treated with GH and those not treated with GH (43). Van den Berg et al. (38) found neither myocardial nor ventricular hypertrophy in a TS population treated with rGH. Smaller LV volumes may reflect cardiac hypoplasia in TS rather than a GH effect (44).

Another interesting finding was that in seven patients (17.9%), pericardial fluid was observed on CMR. Pericardial effusion is rarely described in TS (45, 46). However, past study results were based on echocardiography. In our study, pericardial effusion was recognized on CMR. It can be associated with hypothyroidism and thyroiditis, but in our group, only three out of seven patients had euthyroid Hashimoto's disease. Pericardial effusion and pericarditis may be a sign of systemic inflammatory disease or autoimmune vasculitis. Although none of our patients were diagnosed with systemic disease during the time of the study, a more specific diagnosis should be performed. The increased risk of autoimmune diseases in TS patients has been confirmed (47), and screening for celiac and Hashimoto's diseases or diabetes has become standard practice. The estimation of specific vasculitis antibodies should also be considered in TS.

Improvements in imaging techniques facilitate the diagnosis of cardiovascular anomalies in TS. Most of the cardiovascular anomalies diagnosed on angio-MR were not detected by echocardiography. Additionally, the dilation of the aorta was estimated more precisely with angio-MR. Because of the poor acoustic window and chest wall abnormalities in TS patients, only the AA and aortic arch can be visualized by echocardiography.

## Conclusions

Our study shows that CMR and angio-MR provide a detailed diagnosis of arterial and venous anomalies that have important clinical implications. Magnetic resonance can identify cardiovascular anomalies, dilatation of the aorta, pericardial fluid and functional impairment of ventricles that are missed by echocardiography. BMI, age, BAV, and elongation of the AA influence aortic dilatation. The ASI and AD/DD ratio are important markers of aortic dilatation. The performed diagnostics did not indicate a negative influence of GH treatment on the cardiovascular system. The cardiac measurements made on MRI and echocardiography were comparable in most cases.

## Author contributions

MO-M contributed to the study design, acquisition of data, analysis and interpretation of data, and writing of the manuscript. JR-T contributed to the study design, acquisition of data, analysis and interpretation of data, and critical revision of the manuscript. SR contributed to the acquisition of data, graphics design, and analysis and interpretation of data. AK contributed to the acquisition of data and drafting of the manuscript. KK-K, BR-P, MJ, AG-S, BM, and AS contributed to the acquisition of data. MN and MP contributed to the study design, drafting of the manuscript, and critical revision of the manuscript.

### Conflict of interest statement

The authors declare that the research was conducted in the absence of any commercial or financial relationships that could be construed as a potential conflict of interest.
