# Supplementary material for: The Usefulness of Magnetic Resonance Imaging of the Cardiovascular System in the Diagnostic Work-Up of Patients With Turner Syndrome
Source: Front Endocrinol (Lausanne). 2018 Oct 16;9:609. doi: 10.3389/fendo.2018.00609 (PMC6232706; doi:10.3389/fendo.2018.00609)
Supplement: Supplementary file 1 [file Table_1.DOCX]

| No | Age  [years] | BMI  [kg/m2] | BMI  [centile ] | Height  SDS | Karyotype | Neck  Web  bing  Y/N | GH  therapy  Y/N | Estrogen replace  ment  Y/N | Echo | Cardio  surgery  Y/N | CardioMRI \ AngioMRI | ASI  [mm/m2]  Normal < 2 |
| --- | --- | --- | --- | --- | --- | --- | --- | --- | --- | --- | --- | --- |
| 1 | 21.3 | 26.3 | 94 | -1.3 | 46,X, i(Xq)(q10)  [26]/  45,X[24] | N | Y | Y | Normal | N | Normal | 1.45 |
| 2 | 20.0 | 24.4 | 87 | -3.3 | 45,X | Y | Y | Y | BAV, aortic valve stenosis | N | BAV, aortic valve stenosis,  **common origin LCCA**  **and BCT** | 1.67 |
| 3 | 20.6 | 23.0 | 86 | -2.8 | 45,X[46]/  46,XY[4] | N | Y | N | Normal | N | Normal | 1.63 |
| 4 | 21.7 | 20.2 | 53 | -2.5 | 46,X,i(X)  (q10)[28]/  45,X[22] | N | Y | Y | Normal | N | Normal  **Separate origin of right subclavian artery** | 1.81 |
| 5 | 16.5 | 21.9 | 82 | -2.3 | 45,X[35]/  46Xi(X)  (q10)[15] | Y | Y | y | BAV | Y  CoA | BAV  Normal | 1.57 |
| 6 | 21.8 | 17.9 | 18 | -0.7 | 45,X | Y | Y | Y | Normal | N | **PLSVC,**  **elongation of ascending aorta and aortic arch** | 1.68 |
| 7 | 22.8 | 24.5 | 89 | -3.6 | 45,X[43]/  46,X,i(X)  (q10)[7] | N | Y | Y | BAV | N | BAV,  **elongation of ascending aorta** **and aortic arch** | 1.46 |
| 8 | 22.6 | 21.3 | 37 | -1.3 | 46,X,i(Xq)  (q10)[28]/  45,X[22] | Y | Y | Y | Normal | N | **PAPVR** | 1.36 |
| 9 | 20.3 | 21.9 | 79 | -0.9 | 45,X | Y | Y | Y | BAV | N | BAV | 1.82 |
| 10 | 21.7 | 25.6 | 93 | -1.9 | 45,X | Y | Y | Y | BAV,  dilation of aortic bulb  Hypertension | Y  CoA | BAV.  Mild aortal valve insufficiency.  **PLSVC**  **Aneurysmal dilatation of ascending aorta, coarctation of aorta at left subclavian artery origin,**  **elongation of ascending aorta and aortic arch** | **2.56** |
| 11 | 17.0 | 18.9 | 64 | -2.3 | 45,X[39]/  46,X,i(X)  (q10)[11] | Y | Y | N | Observation of LV and LVOT | N | **Right sided descending aorta**, **pericardial fluid 4 mm, heart arrhythmia,**  **elongation of ascending aorta and aortic arch** | 1.56 |
| 12 | 20.7 | 23.8 | 87 | -0.7 | 45,X[780]/  46,XX [9] | N | Y | N | Normal  Hypertension | N | **Elongation of ascending aorta and aortic arch** | 1.70 |
| 13 | 20.3 | 25.7 | 90 | -2.0 | 45,X[8]/  46,XX[32] | N | Y | N | BAV, aortal valve defect due to hemodyna-  mic disturbances of BAV, observation of ascending aorta | N | BAV, thickening of aortic valve leaflets,  **elongation of ascending aorta and aortic arch** | 1.77 |
| 14 | 21.2 | 21.1 | 68 | -1.2 | 45,X[33]/  46,XX[17] | N | N | Y | Normal | N | **Elongation of ascending aorta and aortic arch** | 1.47 |
| 15 | 16.8 | 21.3 | 88 | -1.0 | 45,X[45]/  46,X,+mar  [5](Y) | Y | Y | N | BAV | N | BAV, **PLSVC drailing into dilated coronary sinus,**  **pericardial fluid 6 mm.** | 1.81 |
| 16 | 20.5 | 21.0 | 71 | -3.1 | 45,X[65]/  46,XY[35] | N | Y | Y | Normal | N | Normal | 1.65 |
| 17 | 22.2 | 20.7 | 54 | -2.8 | 45,X | Y | Y | N | Normal | N | **Elongation of ascending aorta and aortic arch** | 1.97 |
| 18 | 23.3 | 18.3 | 12 | -3.3 | 46,X,i(X)  (q10)[38]/  45,X[12] | N | Y | Y | Normal | N | Normal | 1.76 |
| 19 | 21.4 | 29.7 | 99 | -3.0 | 45,X | Y | Y | Y | Mitral valve insufficiency  I st grade  + MVP,  observation of aortic arch  Hypertension | N | CMR not done/  Normal | 1.52 |
| 20 | 18.4 | 19.2 | 48 | -3.6 | 45,X[49]/  46,XY[1] | N | Y | N | BAV, observation of ascending aorta | Y  CoA | BAV  **Stenosis after surgery not hemodynamically significant,**  **elongation of ascending aorta and aortic arch** | **2.79** |
| 21 | 17.0 | 25.0 | 94 | -2.9 | 45,X[36]/  46,XY[14] | N | Y | Y | Insufficiency of aortic valve | N | **BAV,**  **elongation of ascending aorta** | 1.92 |
| 22 | 13.0 | 13.9 | 10 | -3.7 | 45,X | Y | Y | N | Normal | N | **common origin LCCA and BCT, PAPVR** | **2.13** |
| 23 | 17.5 | 22.8 | 89 | -1.4 | 45,X | Y | Y | Y | After PDA surgery, hemodynamic  BAV, observation of aortic arch | Y  PDA | BAV, **PLSVC, PAPVR, ASD**  **elongation of ascending aorta and aortic arch with stenosis of aorta and poststenotic dilation** | 1.79 |
| 24 | 21.0 | 20.5 | 47 | -0.3 | 45,X[31]/  46,Xi(X)  (q10)[19] | N | Y | Y | Normal | N | Normal | 1.42 |
| 25 | 18. 3 | 32.0 | 99 | -1.1 | 45,X | Y | Y | Y | Normal. | N | **BAV,**  **elongation of ascending aorta and aortic arch** | 1.36 |
| 26 | 15.6 | 18.3 | 60 | -3.5 | 45,X | Y | Y | N | BAV, observation of aortic arch | N | BAV, **PAPVR**  **separate origin of right subclavian artery, elongation of ascending aorta and aortic arch** | 1.73 |
| 27 | 16.1 | 23.5 | 89 | -0.2 | 45,X | N | Y | Y | Normal | N | **Elongation of ascending aorta,**  **origin of the left vertebral artery from aortic arch,**  **pericardial fluid 6 mm** | 1.53 |
| 28 | 13.9 | 23.4 | 93 | -1.7 | 45,X | Y | Y | N | BAV, extension of aortic bulb, insufficiency of mitral valve 2 grade | N | BAV,  **PAPVR ,**  **elongation of ascending aorta and aortic arch** | 1.95 |
| 29 | 15.8 | 18.7 | 62 | -3.0 | 45,X | Y | Y | N | BAV | Y  CoA | BAV  **Coarctation of aorta up to 14 mm** | **2.48** |
| 30 | 14.9 | 17.8 | 44 | -2.8 | 45,X | Y | Y | Y | Normal | N | **BAV, elongation of ascending aorta and aortic arch**  **pericardial fluid 3 mm** | **2.52** |
| 31 | 12.0 | 16.1 | 35 | -3.9 | 45,X | N | Y | N | Normal | N | Normal | **2.47** |
| 32 | 17.9 | 24.4 | 92 | -1.9 | 45,X/  47,XYY | N | Y | Y | BAV | N | **Normal**  **Tricuspid aortic valve.**  angioMR not done | - |
| 33 | 21.2 | 20.3 | 71 | -1.7 | 45,X[40]/  46,X,i(X)  (q10)[10] | Y | Y | y | Normal | N | Normal,  **Pericardium fluid 2 mm** | 1,77 |
| 34 | 19.3 | 23.1 | 86 | -3.6 | 45,X | N | N | N | Observation of aortic valve. | Y  CoA | **Increased trabeculation in left ventricle**  angioMR not done | - |
| 35 | 21.8 | 21.7 | 63 | -2.3 | 46,X,  idic(x)(qter-p11:p11-qter) | N | Y | Y | Normal | N | Normal  angioMR not done | - |
| 36 | 23.8 | 23.0 | 30 | -0.0 | 46,X idic(X)  (p11.2)[47]/45,X[3] | N | N | Y | BAV, aortic valve insufficiency 1st grade | N | BAV, trace insufficiency of the aortic valve | 1.58 |
| 37 | 11.6 | 19.8 | 92 | -1.8 | 47,XXX  [41]/  45,X[9] | N | Y | N | Normal | N | Normal  angioMR not done | - |
| 38 | 17.8 | 22.4 | 88 | -2.7 | 45,X[8]/  46,XX[42] | N | N | N | Normal | N | **Common origin LCCA and BCT pericardial fluid 3 mm** | 1.82 |
| 39 | 15.5 | 22.2 | 88 | -1.5 | 45,X | Y | Y | Y | Normal | N | **PAPVR** | 1.44 |
| 40 | 13.6 | 23.8 | 81 | -2.9 | 46,X+r.ish r(X)(wcpX+)[5/30] | N | Y | N | Normal | N | Normal  angioMR done without contrast | - |
| 41 | 19.3 | 20.0 | 45 | -2.1 | 45,X | Y | Y | Y | Normal | N | **pericardial fluid 4-5 mm**  angioMR not done | - |

**Table S 1.** Clinical characteristics and ECHO, CMR, angio MR results in studied group. In bold are selected clinical significant differences between ECHO and MRI examinations. Legend: BAV bicuspid aortic valve, CoA coarctation of aorta, LV left ventricle, LVOT left ventricular outflow tract, MPV mitral valve prolapsed, PDA patent ductus arteriosus, LCCA left common carotid artery, BCT brachiocephalic trunk, PLSVC persistent left superior vena cava, PAPVR partial anomalous pulmonary venous return.
